# Comparing self-rated health among exclusive e-cigarette users and traditional cigarette smokers: an analysis of the Health Survey for England 2019

**DOI:** 10.1007/s11739-024-03817-y

**Published:** 2024-11-15

**Authors:** Yusuff Adebayo Adebisi, Duaa Abdullah Bafail

**Affiliations:** 1 College of Social Sciences, University of Glasgow, Glasgow, UK; 2 Department of Clinical Pharmacology, Faculty of Medicine, King Abdulaziz University, Jeddah, Saudi Arabia

**Keywords:** E-cigarettes, Traditional cigarette smoking, Self-rated health, Harm reduction, Cross-sectional study

## Abstract

The health implications of e-cigarette use compared to traditional cigarette smoking continue to attract significant public health interest. This study examines self-rated health (SRH) outcomes among exclusive e-cigarette users versus exclusive traditional cigarette smokers, using data from the Health Survey for England 2019. From an initial sample of 10,299 participants, the study focused on 8204 adults, excluding those aged 0–15. Further refinement to exclusive nicotine product users led to 274 e-cigarette users and 1017 cigarette smokers, after excluding dual users, never users, ex-users, non-responders, and users of other tobacco products such as pipes and cigars. SRH was derived from participants’ responses to a question asking how they rated their general health, with five possible options: “very good”, “good”, “fair”, “bad”, and “very bad”. For the purposes of this study, these responses were collapsed into two categories: “Good Health” (combining “very good” and “good”) and “Poor Health” (combining “fair”, “bad”, and “very bad”). Consequently, 834 participants were classified as reporting good health, while 457 reported poor health. Binary logistic regression, adjusted for factors such as age, sex, ethnicity, residence, education, body mass index, alcohol use, age started smoking, physical or mental health conditions, and frequency of GP visits, revealed that exclusive e-cigarette users were significantly more likely to report good health compared to exclusive cigarette smokers, with an odds ratio (OR) of 1.59 (95% CI: 1.10 – 2.32, *p* = 0.014). As a sensitivity analysis, a generalized ordered logistic regression model was performed using the original five SRH categories. The adjusted model confirmed consistent results, with exclusive e-cigarette users showing higher odds of reporting better health across the full range of SRH outcomes (OR = 1.40, 95% CI: 1.08–1.82, *p* = 0.011). These findings suggest that exclusive e-cigarette users perceive their health more positively than traditional cigarette smokers, contributing useful insights to the discussions around harm reduction strategies.

## Introduction

Traditional cigarettes have been unequivocally linked to a myriad of adverse health outcomes, including increased risks of cardiovascular disease, lung cancer, and chronic obstructive pulmonary disease, among others [1–3]. The global effort to curb smoking-related health issues has been a central focus of public health initiatives for several decades, leading to policies aimed at reducing smoking rates through education, regulation, and support for cessation efforts. However, over the past decade, the landscape of nicotine consumption has significantly evolved, marked by the introduction and rapid adoption of electronic cigarettes (e-cigarettes) [4–6]. This new mode of nicotine delivery has reignited debates about nicotine’s health risks, previously well-documented in traditional cigarette smoking [7–9]. Globally, over 82 million people use e-cigarettes [10]. In the UK, including England, electronic cigarettes are permitted, and there are no nationwide legal restrictions or laws specifically enforcing vaping restrictions in public areas [11, 12]. However, many establishments, such as shops, indoor restaurants, trains, and bus shelters, have implemented their own non-vaping policies, resulting in various restrictions across the country.

An expert independent evidence review published by Public Health England concluded that e-cigarettes are around 95% less harmful than smoking [13]. The recent Khan review also advised that to meet the Smokefree 2030 goal (which aims for a smoking prevalence of 5% or less by 2030), the government should expedite the process of making e-cigarettes as a smoking cessation tool [14]. In addition, it recommended offering free vape starter kits to individuals from underprivileged communities. However, the global regulatory framework for e-cigarettes is still evolving [5, 6]. While some countries have embraced e-cigarettes as cessation aids, others have imposed strict regulations due to concerns about long-term health effects and youth uptake [5, 6]. The rapid pace of e-cigarette innovation and market expansion has outstripped the speed of scientific research, leaving gaps in the understanding of their public health impact. E-cigarettes are often presented and perceived as a less harmful alternative to traditional cigarettes, with proponents highlighting their potential role in harm reduction and smoking cessation [5, 15, 16]. For instance, a recent Cochrane review presents strong evidence indicating that e-cigarettes containing nicotine are more effective at helping people quit smoking compared to nicotine replacement therapies and e-cigarettes without nicotine [17]. Despite this, the health implications of long-term e-cigarette use remain largely uncertain. The dichotomy between traditional smoking and e-cigarette use has, thus, become a critical area of investigation, necessitating a deeper understanding of how these two methods of nicotine consumption compare in terms of health outcomes.

Exploring self-rated health (SRH) in e-cigarette users compared to cigarette smokers is crucial, as SRH has emerged as a valuable indicator in public health research, reflecting individuals’ perceptions of their own health status [18–20]. SRH encompasses a broad range of physical, psychological, and social well-being dimensions and has been shown to be a reliable predictor of morbidity and mortality [19, 20]. Investigating SRH among e-cigarette users and traditional smokers can provide insights into how these groups perceive their health, potentially revealing the impacts of different nicotine delivery systems on overall health perceptions. The subjective nature of SRH allows for the capture of health nuances that objective measures might overlook, offering a view of an individual’s health that encompasses both medical conditions and the perception of well-being [21]. This is particularly important in the context of e-cigarettes, where the perception of risk and harm may significantly influence users’ health behaviors and choices.

SRH also aligns with the Biopsychosocial Model, which was proposed by George Engel [22]. It allows individuals to assess their health based on personal perceptions and experiences, capturing the subjective nuances of health that traditional medical assessments may overlook [23–25]. This approach acknowledges that individuals’ perceptions of their health are shaped by a complex mix of factors, including their biological conditions, psychological resilience, and the social contexts in which they live and interact. Currently, little is known about how self-rated health compares between adult current smokers and current e-cigarette users. Given this context, the present research seeks to fill a gap in the literature by comparing SRH among exclusive e-cigarette users and traditional cigarette smokers. This comparison is timely and relevant, considering the shifting landscape of nicotine consumption and the ongoing debates about the role of e-cigarettes in public health.

## Method

### Study design, participants, and data source

This cross-sectional study utilized data from the Health Survey for England (HSE) 2019, the most recent dataset of the HSE series that is publicly available. The HSE is an invaluable resource for both central and local government entities, such as the Department of Health & Social Care and Public Health England [26]. It serves multiple functions, including monitoring shifts in health and lifestyle patterns, determining the prevalence of specific health conditions, aiding in service planning, informing policy development, and evaluating the impacts of health policies [26]. The 2019 survey, conducted by NatCen Social Research on behalf of NHS England, utilized a multi-stage stratified randomized selection process for addresses to ensure the sample accurately represented individuals living in private households across England [27]. Throughout the year, from January to December 2019, a total of 9612 addresses were selected from 534 postcode sectors for the survey, with fieldwork completed by March 2020 [28]. When an address included multiple dwelling units or households, one of each was randomly chosen for inclusion in the study. Now in its twenty-ninth year, the HSE 2019 dataset is accessible through the UK Data Service website, and the specific methodologies used are detailed in other documents [28].

The original dataset included 10,299 participants, spanning adults aged 16 and above as well as children aged 0–15. For our analysis, we concentrated solely on the adult population, excluding the 2095 children from our dataset. This adjustment resulted in a focused sample size of 8204 adult participants for further exclusion.

### Assessment of the main exposures: current cigarette smoking and current e-cigarette usage

The primary exposures in our study were current cigarette smoking and current e-cigarette usage, specifically among individuals who either exclusively use e-cigarettes or exclusively smoke cigarettes. To categorize participants according to their exposure status, we used 2 variables from the dataset, which included 8204 participants (after excluding children): cigsta3_19 and eciguse_19. The cigsta3_19 variable classified respondents into current cigarette smokers (*n* = 1254), ex-regular cigarette smokers (*n* = 2076), never regular cigarette smokers (*n* = 4818), with some respondents either refusing to answer (*n* = 24), not knowing (*n* = 13), or being categorized as not applicable (*n* = 19). Similarly, the eciguse_19 variable divided respondents into current e-cigarette users (*n* = 471), ex-regular e-cigarette users (*n* = 1109), and never regular e-cigarette users (*n* = 6579), with additional responses for those who refused (*n* = 24), did not know (*n* = 2), or were not applicable (*n* = 19). From these data, we derived a new variable with four categories: exclusive e-cigarette users (*n* = 281), exclusive cigarette smokers (*n* = 1066), dual users (*n* = 188), and a combined ‘others’ category (*n* = 6669), which included ex-smokers, never smokers, never e-cigarette users, ex-e-cigarette users, and non-respondents. In our analysis, which focused on the primary exposure of exclusive use of either product, we excluded dual users and the ‘others’ category, resulting in a binary exposure variable composed of exclusive e-cigarette users (*n* = 281) and exclusive cigarette smokers (*n* = 1066). To isolate the relationship with the outcome variable more accurately, participants currently using cigars and pipes (*n* = 56) were excluded, yielding final variables for exclusive e-cigarette users (*n* = 274) and exclusive cigarette smokers (*n* = 1017).

In our analytical approach, we prioritized the accurate classification of participants based on their primary exposure—current cigarette smoking or current e-cigarette usage—before assessing the outcome of self-rated health. This sequence in exclusion criteria ensured that the analysis was focused solely on individuals with a clear and exclusive use of either product, thereby eliminating confounding factors associated with dual use or non-standard nicotine consumption methods such as cigars and pipes.

### Assessment of the main outcome: self-rated general health

The outcome variable of interest was self-rated general health, derived from the GenHelf variable in the dataset, based on the question: “How is your health in general? Would you say it was…READ OUT… 1 …very good, 2 good, 3 fair, 4 bad, or 5 very bad?”. This categorized health into very good (*n* = 272), good (*n* = 562), fair (*n* = 277), bad (*n* = 135), and very bad (*n* = 45). We consolidated these responses into a binary variable, labeling “very good” and “good” as “Good Health” and “fair”, “bad”, and “very bad” as “Poor Health”. This reclassification, which has been widely used in the literature [29–31], resulted in two distinct groups for further analysis: those reporting good health (*n* = 834) and those reporting poor health (*n* = 457). This binary classification was applied to simplify interpretation and ensure sufficient sample sizes in each group for meaningful comparisons.

### Information on other covariates

In addition to the primary exposures of current cigarette smoking and current e-cigarette usage, our study also considered a comprehensive set of covariates to control for potential confounding factors. These included age group, sex, ethnicity, residence, education level, body mass index (BMI), alcohol consumption frequency, the presence of longstanding physical or mental conditions, age started smoking and frequency of general practitioner (GP) visits in the last 12 months. Age started smoking was categorized into early starters (≤ 16 years) and late starters (> 16 years) to capture the potential long-term effects of smoking exposure. Age group was categorized to reflect different life stages that might impact health behaviors and outcomes. Sex and ethnicity were included to account for demographic differences in health status and access to healthcare. Residence and education level provided a socio-economic context, reflecting environmental and knowledge-based influences on health. BMI was categorized based on World Health Organization standards to assess the impact of weight status on health: underweight (BMI < 18.5 kg/m^2^), normal weight (BMI 18.5–24.9 kg/m^2^), overweight (BMI 25–29.9 kg/m^2^), and obese (BMI ≥ 30 kg/m^2^). Alcohol consumption frequency, longstanding health conditions, and GP visit frequency were considered as they represent lifestyle choices and health engagement that could significantly affect self-rated health. Missing and unreported values in the covariates were classified in a separate category.

### Statistical analyses

Descriptive statistics summarized participant characteristics, using frequencies and percentages for categorical variables. We conducted Chi-square tests to explore differences in categorical variables between exclusive e-cigarette users and exclusive cigarette smokers. In our primary analysis, binary logistic regression models were utilized to calculate odds ratios (ORs) and 95% confidence intervals (CIs) for the relationship between nicotine product use and self-rated health. Covariates were adjusted for in the model regardless of their significance in bivariate analysis; this approach was taken to control for potential confounding factors, ensuring a comprehensive analysis based on the theoretical underpinnings of the study. The covariates adjusted for included age group, sex, ethnicity, residence, education level, BMI, alcohol consumption frequency, age started smoking, the presence of longstanding physical or mental conditions, and the frequency of GP visits in the last 12 months. Both crude and adjusted ORs were reported to illustrate the impact of these adjustments. To assess multicollinearity, we employed the variance inflation factor (VIF) after fitting the final regression model. In addition, to evaluate the predictive validity of the binary logistic regression model, we performed an analysis comparing the mean apparent receiver operating characteristic area under the curve (ROC AUC) with the mean cross-validated ROC AUC using tenfold cross-validation. This comparison helps determine the model’s generalizability and assess the potential for overfitting. In addition, we conducted a Hosmer–Lemeshow goodness-of-fit test to assess how well the model’s predicted probabilities matched the observed outcomes.

A sensitivity analysis was conducted to further assess the robustness of the results by reversing the coding of the variable ‘GenHelf’ for interpretability purposes. In this reversed coding, self-rated health was transformed to range from 1 (Very Bad) to 5 (Very Good), so that higher values corresponded to better health outcomes. Initially, an unadjusted ordered (ordinal) logistic regression (ologit) model was employed to assess the crude association, assuming proportional odds. Subsequently, an adjusted ordered logistic regression model was planned to examine the association while controlling for age group, sex, ethnicity, residence, education level, BMI, alcohol consumption frequency, age started smoking, the presence of longstanding physical or mental conditions, and frequency of GP visits. If the proportional odds assumption was violated in the adjusted model, a generalized ordered logistic regression (gologit2) model with the autofit option would be applied to relax the proportional odds assumption as needed.

A *p* value threshold of 0.05 was adopted to determine statistical significance. All statistical analyses were conducted using STATA version 18.

## Results

We analyzed data from 1291 participants, comprising 1017 exclusive cigarette smokers and 274 exclusive e-cigarette users. Our findings, detailed in Table 1, reveal significant variations in participant characteristics across several demographic and health-related variables.

**Table 1:** Participant characteristics by exclusive traditional cigarette smokers and exclusive e-cigarette users

Variable	Exclusive current cigarette smokers (*n* = 1017)	Exclusive e-cigarette users (*n* = 274)	All (*n* = 1291)	χ2, *p* value
Age group, *n* (%)				χ2 = 17.32, *p* = 0.008*
16–24	123 (12.1)	20 (7.3)	143 (11.1)	
25–34	213 (20.9)	57 (20.8)	270 (20.9)	
35–44	187 (18.4)	58 (21.2)	245 (18.9)	
45–54	182 (17.9)	64 (23.4)	246 (19.1)	
55–64	165 (16.2)	53 (19.3)	218 (16.9)	
65–74	100 (9.8)	13 (4.7)	113 (8.8)	
75 +	47 (4.6)	9 (3.3)	56 (4.3)	
Sex, *n* (%)				χ2 = 2.21, *p* = 0.137
Male	472 (46.4)	141 (51.5)	613 (47.5)	
Female	545 (53.6)	133 (48.5)	678 (52.5)	
Ethnicity, *n* (%)				χ2 = 3.45, *p* = 0.063
White	917 (90.2)	257 (93.8)	1,174 (90.9)	
Non-White	100 (9.8)	17 (6.2)	117 (9.1)	
Residence, *n* (%)				χ2 = 0.40, *p* = 0.527
Urban	887 (87.2)	235 (85.8)	1,122 (86.9)	
Rural	130 (12.8)	39 (14.2)	169 (13.1)	
Education level, *n* (%)				χ2 = 19.89, *p* < 0.001*
Higher qualification	173 (17.0)	57 (20.8)	230 (17.8)	
Below degree	540 (53.1)	172 (62.8)	712 (55.2)	
No qualification	304 (29.9)	45 (16.4)	349 (27.0)	
Body mass index, *n* (%)				χ2 = 17.22, *p* = 0.002*
Underweight	24 (2.4)	1 (0.4)	25 (1.9)	
Normal	290 (28.5)	59 (21.5)	349 (27.0)	
Overweight	302 (29.7)	85 (31.0)	387 (30.0)	
Obesity	209 (20.6)	82 (29.9)	291 (22.5)	
Alcohol consumption, *n* (%)				χ2 = 2.56, *p* = 0.277
Frequent drinker	472 (46.5)	136 (49.6)	608 (47.1)	
Occasional drinker	358 (35.2)	99 (36.1)	457 (35.4)	
Non-drinker	186 (18.3)	39 (14.2)	225 (17.4)	
Age started smoking, *n* (%)				χ2 = 4.74, *p* = 0.029*
Early starters (≤ 16 years)	579 (56.9)	176 (64.2)	755 (58.5)	
Late starters (> 16 years)	438 (43.1)	98 (35.8)	536 (41.5)	
Longstanding physical or mental conditions, *n* (%)				χ2 = 0.000, *p* = 0.995
Yes	486 (47.8)	131 (47.8)	617 (47.8)	
No	531 (52.2)	143 (52.2)	674 (52.2)	
GP visits in the last 12 months, *n* (%)				χ2 = 3.34, *p* = 0.189
No GP visit	293 (28.8)	67 (24.5)	360 (27.9)	
One to two times	304 (29.9)	96 (35.0)	400 (31.0)	
Three or more times	419 (41.2)	111 (40.5)	530 (41.1)	
Self-rated health (binary), *n* (%)				χ2 = 5.85, *p* = 0.016*
Poor health	377 (37.1)	80 (29.2)	457 (35.4)	
Good health	640 (62.9)	194 (70.8)	834 (64.6)	
Self-rated health (ordinal), *n* (%)				χ2 = 7.08, *p* = 0.132
Very Bad	39 (3.8)	6 (2.2)	45 (3.5)	
Bad	114 (11.2)	21 (7.7)	135 (10.5)	
Fair	224 (22.0)	53 (19.3)	277 (21.5)	
Good	432 (42.5)	130 (47.5)	562 (43.5)	
Very Good	208 (20.5)	64 (23.4)	272 (21.1)	

Statistically significant *p* value < 0.05

Age distribution among participants showed significant differences (χ2 = 17.32, *p* = 0.008), with a lower percentage of the younger age group (16–24 years) among e-cigarette users (7.3%) compared to traditional smokers (12.1%). Education level also varied significantly (χ2 = 19.89, *p* < 0.001), with a higher proportion of e-cigarette users (62.8%) having below degree-level education compared to cigarette smokers (53.1%). Notably, the analysis of body mass index categories indicated significant differences (χ2 = 17.22, *p* = 0.002), with a higher proportion of obesity among e-cigarette users (29.9%) compared to cigarette smokers (20.6%). Despite these significant demographic and health-related differences, there was no significant variation in sex (χ2 = 2.21, *p* = 0.137), ethnicity (χ2 = 3.45, *p* = 0.063), and residence (urban vs. rural, χ2 = 0.40, *p* = 0.527). In addition, alcohol consumption habits and the presence of longstanding physical or mental conditions did not differ significantly between the groups, nor did the frequency of GP visits in the last 12 months. Notably, a higher proportion of e-cigarette users (64.2%) started smoking at an early age (≤ 16 years), compared to cigarette smokers (56.9%) (χ2 = 4.74, *p* = 0.029). Interestingly, our analysis revealed a significant relationship between the type of nicotine product used and self-rated health (χ2 = 5.85, *p* = 0.016), with a higher proportion of e-cigarette users (70.8%) reporting good health than traditional smokers (62.9%), and fewer e-cigarette users (29.2%) indicating poor health compared to smokers (37.1%).

### Association with self-rated health

In Table 2, we report the crude and adjusted associations between exclusive e-cigarette use and exclusive traditional cigarette smoking with self-rated health. In the unadjusted model (Model 1), exclusive e-cigarette users had 43% higher odds of reporting good health compared to exclusive cigarette smokers (OR = 1.43, 95% CI: 1.07 – 1.90, *p* = 0.016). After adjusting for age group and sex in Model 2, the odds slightly increased (OR = 1.44, 95% CI: 1.07 – 1.95, *p* = 0.017). Further adjustments in Model 3 for residence, educational level, and ethnicity reduced the odds ratio to 1.31 (95% CI: 0.97 – 1.79, *p* = 0.081), indicating a reduction in the effect size when broader demographic factors were considered. In Model 4, with the additional adjustment for body mass index, the odds ratio slightly increased again to 1.37 (95% CI: 1.00 – 1.87, *p* = 0.049), indicating a borderline significant association between exclusive e-cigarette use and good self-rated health. Model 5, which further accounted for alcohol consumption frequency and age started smoking, resulted in an odds ratio of 1.42 (95% CI: 1.03 – 1.94, *p* = 0.033), maintaining statistical significance.

**Table 2:** Crude and adjusted association between exclusive current cigarette smokers vs. exclusive e-cigarette users and self-rated health

Model	Odd ratio (95% CI), *p* value
Model 1 (unadjusted/crude)	
Exclusive e-cigarette users	1.43 (1.07 – 1.90), *p* = 0.016
Exclusive cigarette smokers	Reference
Model 2 (adjusted for age group and sex)	
Exclusive e-cigarette users	1.44 (1.07 – 1.95), *p* = 0.017
Exclusive cigarette smokers	Reference
Model 3 (adjusted for age group, sex, residence, educational level, and ethnicity)	
Exclusive e-cigarette users	1.31 (0.97 – 1.79), *p* = 0.081
Exclusive cigarette smokers	Reference
Model 4 (adjusted for age group, sex, residence, educational level, ethnicity, and body mass index)	
Exclusive e-cigarette users	1.37 (1.00 – 1.87), *p* = 0.049
Exclusive cigarette smokers	Reference
Model 5 (adjusted for age group, sex, residence, educational level, ethnicity, body mass index, alcohol consumption frequency and age started smoking)	
Exclusive e-cigarette users	1.42 (1.03 – 1.94), *p* = 0.033
Exclusive cigarette smokers	Reference
Final model (adjusted for age group, sex, residence, educational level, ethnicity, body mass index, alcohol consumption frequency, age started smoking and presence of longstanding physical or mental conditions and GP visits)	
Exclusive e-cigarette users	1.59 (1.10 – 2.32), *p* = 0.014
Exclusive cigarette smokers	Reference
Final model characteristics	Parameter
McFadden’s pseudo R-squared	33%
Model fitness (likelihood ratio Chi-square and *p* value)	χ2 = 554.94, *p* < 0.001

Reference outcome: poor health; statistically significant *p* value < 0.05

The final model, comprehensively adjusted for age group, sex, residence, educational level, ethnicity, body mass index, alcohol consumption frequency, age started smoking, the presence of longstanding physical or mental conditions, and GP visits, demonstrated a stronger association (OR = 1.59, 95% CI: 1.10 – 2.32, *p* = 0.014). This suggests that exclusive e-cigarette users are significantly more likely to report good health compared to exclusive cigarette smokers when a wide range of demographic and health-related factors are controlled for. The progression of models shows that although adjustments for covariates affect the strength of the association between nicotine product use and self-rated health, exclusive e-cigarette users consistently report better health outcomes than exclusive cigarette smokers. The robustness of the final model is supported by a McFadden’s pseudo R-squared value of 33% and a highly significant model fit (χ2 = 554.94, *p* < 0.001).

**Fig. 1:**
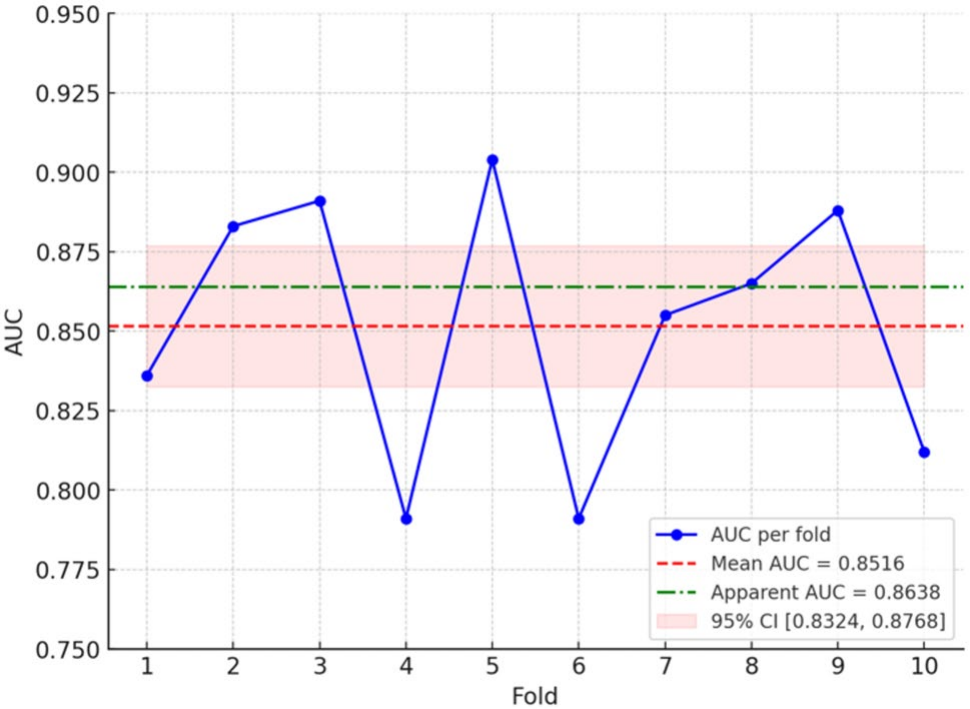
Cross-validated and apparent ROC AUC for the logistic regression model

To evaluate the performance of the binary logistic regression in the final model, we assessed both its goodness-of-fit and predictive validity. The Hosmer–Lemeshow goodness-of-fit test indicated that the model fit the data well (χ^2^(8) = 10.67, *p* = 0.2209), demonstrating that there is no significant difference between the observed and predicted probabilities, and thus the model provides a satisfactory fit. To assess multicollinearity, the mean VIF was 3.34, with individual VIFs ranging from 1.19 to 9.52, indicating mild multicollinearity but below the critical threshold (VIF > 10), suggesting no major concerns for most predictors. To assess the predictive validity of the binary logistic regression model, we calculated both the apparent and cross-validated area under the receiver operating characteristic curve (AUC). The apparent AUC was 0.8638, while the mean cross-validated AUC from a tenfold cross-validation was 0.8516 (95% CI: 0.8324–0.8768), with a standard deviation of 0.0422 (See Fig. 1). The small difference between the apparent and cross-validated AUCs (Δ = 0.0122) indicates that the model generalizes well and is not overly optimistic or overfitted. These results suggest that the model has strong predictive performance, both when assessed on the training data and when applied to unseen data.

### Sensitivity analysis result

In the unadjusted analysis, an ordered logistic regression (ologit) model was employed to examine the association between exclusive e-cigarette use and exclusive traditional cigarette smoking with self-rated health, assuming proportional odds. The likelihood-ratio test indicated no violation of the proportional odds assumption (*p* = 0.5912), confirming that the relationship was consistent across categories. The model showed that exclusive e-cigarette users were significantly more likely to report better self-rated health compared to exclusive cigarette smokers. The odds ratio (OR) for reporting better health was 1.34 (95% CI: 1.05–1.71, *p* = 0.018), suggesting that exclusive e-cigarette users were 34% more likely to rate their health higher than smokers across the full range of the self-rated health scale (See Table 3).

**Table 3:** Ordinal logistic regression results for self-rated health (Very Bad to Very Good) comparing exclusive e-cigarette users and cigarette smokers

Model, *n* = 1291	Odd ratio (95% CI), *p* value	McFadden’s pseudo R-squared ( likelihood ratio Chi-square and *p* value)
Model 1 (unadjusted/crude)		0.16% (χ2 = 5.61 and *p* = 0.018)
Exclusive e-cigarette users	1.34 (1.05 – 1.71), 0.018	
Exclusive cigarette smokers	Ref	
Model 2 (fully adjusted)*		19.8% (χ2 = 702.45 and *p* < 0.001)
Exclusive e-cigarette users	1.40 (1.08–1.82), 0.011	
Exclusive cigarette smokers	Ref	

Outcome: Very Bad is coded as 1, Bad as 2, Fair as 3, Good as 4 and Very Good as 5

Statistically significant *p* value < 0.05

*Adjusted for age group, sex, residence, educational level, ethnicity, body mass index, alcohol consumption frequency, age started smoking and presence of longstanding physical or mental conditions and GP visits

After controlling for potential confounders, including age group, sex, ethnicity, residence, education level, BMI, alcohol consumption frequency, age started smoking, longstanding illness, and frequency of GP visits, the adjusted ordered logistic regression model revealed a significant violation of the proportional odds assumption (*p* = 0.007). Due to this violation, a generalized ordered logistic regression (gologit2) model was used to relax the proportional odds assumption where necessary. In this adjusted gologit2 model, exclusive e-cigarette users were 40% more likely to report better overall health compared to exclusive cigarette smokers (OR = 1.40, 95% CI: 1.08–1.82, *p* = 0.011). These results, similar to those from the binary logistic regression, indicate that exclusive e-cigarette use is associated with a more favorable self-assessment of health relative to exclusive cigarette smoking, underlining the robustness of the association across different modeling approaches.

## Discussion

In this study, we investigated self-rated health among exclusive e-cigarette users compared to exclusive cigarette smokers, providing insights into subjective health perceptions among users of different nicotine delivery systems. Our findings revealed that exclusive e-cigarette users were significantly more likely to report good health compared to exclusive cigarette smokers, even after adjusting for multiple demographic and health-related covariates in a binary logistic regression model. Sensitivity analyses using a generalized ordered logistic regression model with the original five-category self-rated health variable confirmed these results, demonstrating a stable and reliable association across modeling approaches. This aligns with previous findings where both dichotomous and ordinal models yielded comparable results in analyzing self-rated health [30]. Together, these results suggest that the relationship between e-cigarette use and better self-rated health is robust, minimizing concerns about model instability. This observation is particularly important as it reflects a broader trend noted in public health research, where e-cigarettes have been positioned as a potentially less harmful alternative to traditional cigarettes [32–35].

The narrative that e-cigarettes may contribute to harm reduction, as suggested by evidence including the Public Health England review, supports our findings that users of e-cigarettes perceive their health more positively [13]. Recent data from Action on Smoking and Health further reinforce this, showing that e-cigarettes have been the most popular aid for quitting smoking in the UK over the past 5 years, with 2.7 million people using them to quit [15]. Moreover, population-level studies have shown that e-cigarettes are also associated with lower smoking prevalence across populations [34, 35]. This overall decline in smoking rates may lead to improved health outcomes and perceptions, as fewer individuals are exposed to the harmful effects of traditional cigarettes. Consequently, the positive self-rated health among exclusive e-cigarette users observed in our study may reflect these broader public health trends.

While self-rated health offers useful insights into personal health perceptions, objective measures provide a more concrete assessment of the physiological impact of e-cigarette use. Several studies have reported potential cardiovascular and respiratory benefits for smokers who switch to e-cigarettes. For instance, research has shown that smokers who transition to e-cigarettes experience improvements in cardiovascular parameters such as reductions in systolic blood pressure, endothelial function improvements, and reduction in arterial stiffness [36–40]. These findings suggest that e-cigarettes may help mitigate some of the cardiovascular risks associated with traditional smoking, at least in the short term. Similarly, in terms of respiratory health, studies have noted improvements in lung function and pulmonary ventilation and decreases in airway resistance among smokers who switched to e-cigarettes [41, 42], pointing to a potential benefit in reducing the harm caused by tobacco smoke.

In addition to cardiovascular and respiratory improvements, a recent systematic review and meta-analysis found that exposure to tobacco-specific nitrosamines (TSNAs)—potent carcinogens found in tobacco smoke—was significantly lower among exclusive e-cigarette users compared to exclusive cigarette smokers [43]. The study reported substantial reductions in TSNA levels when individuals switched from smoking to e-cigarettes, indicating decreased exposure to harmful carcinogens. This reduction in carcinogen exposure may potentially lower the risk of cancer and other diseases linked to TSNAs, such as cardiovascular and respiratory illnesses, among e-cigarette users compared to traditional smokers. Such objective improvements in health risk profiles may enhance individuals’ self-rated health, as they recognize the benefits of reduced exposure to harmful substances. The psychological reassurance of making a healthier choice and reducing one’s risk of serious illnesses could contribute to the higher self-rated health reported by exclusive e-cigarette users in our study.

On the other hand, not all studies have reported such benefits. Some research has found no significant improvements in cardiovascular or respiratory markers, and in some cases, switching to e-cigarettes has been associated with negative impacts on these markers and other objective parameters [45–48]. These mixed results also suggest that the potential benefits of e-cigarettes on health may vary depending on individual factors such as the duration of smoking, baseline health status, or the specific type of e-cigarette used. While the evidence is indeed mixed, with some studies showing clear benefits and others demonstrating no benefits, the harm reduction effect of e-cigarettes compared to traditional smoking is widely acknowledged. By eliminating combustion, which is responsible for many of the toxic substances in cigarette smoke, e-cigarettes offer a less harmful alternative. Nonetheless, it is important to continue gathering objective data, particularly through long-term studies, to fully assess the benefits and potential risks of e-cigarette use in comparison to traditional smoking.

Despite the reliance on subjective measures in our study, it remains relevant in contributing to ongoing debates about nicotine use and harm reduction. Self-rated health is widely recognized in public health as a useful indicator of how individuals view their own well-being, which can inform broader understanding of health behaviors and interventions [18, 49]. Although it may not provide direct evidence of harm reduction, the fact that e-cigarette users report better perceived health than smokers is still meaningful, especially in contexts where public health messaging strongly promotes e-cigarettes as a safer alternative. Importantly, this study aligns with previous research suggesting that individuals may perceive e-cigarettes as less harmful [50, 51], contributing to harm reduction frameworks that aim to reduce the adverse impacts of smoking on populations. Thus, while self-reported health status does not equate to medical or long-term health outcomes, the perceptions captured in this study offer insight into the personal experiences of nicotine product users, which remains significant for public health policies and harm reduction strategies. Contextually, the shift toward e-cigarettes as an alternative to traditional cigarettes has grown, with over 82 million users worldwide, many of whom have switched from traditional smoking [10, 52]. Understanding how these individuals perceive their health post-switch is pertinent, as self-rated health is often used to gauge immediate health perceptions. Public health strategies should take these subjective perceptions into account, alongside objective evidence, to guide interventions aimed at promoting smoking cessation and harm reduction. Although public health authorities continue to explore the full health implications of e-cigarettes, studies like this one provide valuable insights into user experiences, which can influence behavior and inform public health policy.

This study’s strength lies in its comprehensive analysis of self-rated health outcomes among exclusive e-cigarette users and cigarette smokers, utilizing a large, representative sample from the Health Survey for England 2019. Our inclusion of key demographic and health-related covariates ensures that our findings are adjusted for factors such as age, sex, education, and health conditions, lending credibility to the comparisons drawn between these two groups. Moreover, this study fills a gap in the literature by focusing on exclusive users, providing insights into the health perceptions of individuals who engage in distinct nicotine consumption behaviors without the confounding effects of dual use.

However, several limitations must be acknowledged. The reliance on self-reported data introduces potential biases, as individuals may be influenced by external factors such as public health messaging that positions e-cigarettes as a healthier alternative. This perception bias, which is particularly strong in the UK, could have led e-cigarette users to report better health than cigarette smokers, regardless of their actual health status. Nonetheless, the consistency of the self-reported health data with the harm reduction narrative widely supported in the literature suggests that this bias may not have significantly affected the overall findings. In addition, residual confounding due to unmeasured variables cannot be ruled out, which may influence the observed associations. While this study adjusted for age started smoking—an important factor in understanding early nicotine exposure—it did not fully account for the duration of nicotine use. Duration is important, as long-term and short-term users of either nicotine product may experience different health trajectories [53]. Future research should aim to capture cumulative nicotine exposure over time, which may provide a more complete picture of health outcomes. Despite these limitations, the adjustments for demographic and health-related covariates help ensure that the findings provide insights into general health perceptions among nicotine users. Although small cell sizes in certain subgroups, such as underweight e-cigarette users, may reduce the precision of estimates for these categories, the overall sample size and model performance remain robust.

The cross-sectional design further limits our ability to infer causality or long-term health outcomes, as self-rated health at a single point in time does not capture changes in health over extended periods [54, 55]. However, the associations observed provide an important snapshot of health perceptions, which are highly relevant for public health messaging and interventions, particularly in understanding the immediate impacts of switching to e-cigarettes. Finally, the exclusion of participants under 16 years of age limits the generalizability of these findings to the entire population of nicotine users. Nonetheless, focusing on adult populations is particularly relevant, as they represent the majority of e-cigarette and traditional cigarette users, making the findings highly applicable to key target groups in harm reduction strategies.

Future research should focus on addressing the limitations identified in this study. First, longitudinal studies are needed to track health outcomes over time, allowing researchers to examine the long-term effects of exclusive e-cigarette use versus traditional smoking. This would provide stronger evidence for or against the harm reduction potential of e-cigarettes. In addition, the duration of nicotine product use should be factored into future analyses, as long-term exposure may present different health risks or benefits compared to short-term use. Exploring the role of social messaging in shaping health perceptions could also offer valuable insights into how public health campaigns influence behavior and self-reported health. Expanding the scope of research to include underage users, dual users, and individuals who use other nicotine products (e.g., heated tobacco or nicotine pouches) would provide a more comprehensive understanding of nicotine consumption patterns and their health impacts. Finally, combining subjective measures like self-rated health with objective health outcomes (e.g., respiratory function tests or cardiovascular assessments) would create a more holistic view of the health implications of e-cigarettes, ultimately guiding more effective harm reduction and smoking cessation strategies.

## Conclusion

Our findings suggest that exclusive e-cigarette users are more likely to perceive better health compared to traditional cigarette smokers, even after adjusting for demographic and health-related factors. This highlights the potential of e-cigarettes as a harm reduction tool. However, given that these findings are based on self-rated health, a subjective measure, they should be interpreted cautiously. They reflect perceived health benefits rather than definitive evidence of actual health outcomes or safety. To better understand the long-term implications of e-cigarette use, comprehensive longitudinal studies are necessary, particularly those incorporating objective health measures. While our study contributes valuable insights to the discourse on harm reduction, it underlines the importance of further research to fully assess the public health impact of e-cigarettes. In addition, public health messaging should strive for balance, accurately communicating both the potential risks and benefits of e-cigarette use to inform user choices effectively.

## Data Availability

To download the dataset used in the analyses, please visit the https://ukdataservice.ac.uk/find-data/browse/health/.

## Declarations

### Patient and public involvement

Patients and/or the public were not involved in the design, or conduct, or reporting, or dissemination plans of this research.
